# Homozygous DHCR7 p.Val330Met Variant Associated with Mild Non-Syndromic Intellectual Disability and Elevated Serum 7-Dehydrocholesterol Levels in Two Siblings

**DOI:** 10.3390/genes16070838

**Published:** 2025-07-18

**Authors:** Lukas Hackl, Edda Haberlandt, Thomas Müller, Susanne Piribauer, Dorota Garczarczyk-Asim, Thomas Zöggeler, Daniela Karall, Johannes Zschocke, Andreas R. Janecke

**Affiliations:** 1 Department of Paediatrics I, Medical University of Innsbruck, 6020 Innsbruck, Austria; lukas.hackl@tirol-kliniken.at (L.H.); thomas.mueller@tirol-kliniken.at (T.M.); dorota.garczarczyk@i-med.ac.at (D.G.-A.); thomas.zoeggeler@tirol-kliniken.at (T.Z.); daniela.karall@i-med.ac.at (D.K.); 2 Krankenhaus der Stadt Dornbirn, Kinder- und Jugendheilkunde, 6850 Dornbirn, Austria; edda.haberlandt@dornbirn.at (E.H.); susanne.piribauer@dornbirn.at (S.P.); 3 Institute of Human Genetics, Medical University of Innsbruck, 6020 Innsbruck, Austria; johannes.zschocke@i-med.ac.at

**Keywords:** 7-dehydrocholesterol reductase, cholesterol metabolism, congenital malformation syndrome, DHCR7, genotype-phenotype correlation, Smith-Lemli-Opitz syndrome

## Abstract

Biallelic pathogenic variants in *DHCR7* result in decreased activity of 7-dehydrocholesterol (7-DHC) reductase, which converts 7-DHC to cholesterol, and causes Smith–Lemli–Opitz syndrome (SLOS). Elevated serum 7-DHC levels are indicative of SLOS as are intellectual disability (ID), growth retardation, microcephaly, craniofacial anomalies, and 2–3 toe syndactyly. Additional congenital malformations may be present in SLOS, and broad clinical variability has been recognized in SLOS. Rarely, biallelic pathogenic *DHCR7* variants were reported with low-normal and normal intelligence quotient (IQ) and development. We report here a pair of siblings with mild global developmental delay, infrequent epileptic seizures, and elevated serum 7-DHC levels, associated with the homozygous *DHCR7* variant c.988G>A (p.Val330Met). Remarkably, neither sibling displayed congenital anomalies nor dysmorphisms. Quattro-exome sequencing performed for global delay and mild ID in both siblings did not identify other ID causes. c.988G>A affects a highly conserved amino acid and displays a relatively high global population allele frequency of 0.04%, with absence of homozygotes from the population database gnomADv4.1.0. Our observation leads us to suggest that *DHCR7* variant c.988G>A and other *DHCR7* variants might be generally considered as underlying non-syndromic ID.

## 1. Introduction

*DHCR7* encodes 7-dehydrocholesterol (7-DHC) reductase, the enzyme that catalyzes the final step in cholesterol biosynthesis [1]. Deficiency in 7-DHC reductase typically leads to low levels of cholesterol and increased levels of precursors 7-DHC and 8-DHC in blood and tissues [2]. Biallelic *DHCR7* variants cause the autosomal recessive Smith–Lemli–Opitz syndrome (SLOS, OMIM #270400) [3,4,5,6,7]. Typical SLOS manifestations are present in most patients and comprise intellectual disability (ID), growth retardation, minor craniofacial anomalies, microcephaly, and 2–3 toe syndactyly. A spectrum of congenital malformations is variably present [8,9]. The clinical spectrum spans from fetal to neonatal death to reportedly normal phenotypes [10,11,12,13]. Typically, individuals with SLOS present moderate to severe ID, but low-normal and borderline-normal cognitive functioning in SLOS has been reported in some cases [13,14,15]. Behavioral abnormalities were present in >90% of individuals with SLOS and include sleep disturbances, irritability, repetitive and ritualistic behaviors, aggressiveness, self-injury, social and communication impairment, attention deficit hyperactivity disorder, and autism spectrum disorder [15,16,17]. A correlation has been reported between cholesterol and 7-DHC levels and a severity score based on congenital malformations with developmental outcomes [7,18]. Malformation in SLOS results from a cholesterol deficit during embryogenesis, which impairs activation of the sonic hedgehog signaling component smoothened (SMO) and its localization to the primary cilium [19]. The most severe SLOS phenotypes result from two *DHCR7* null variants, and can still vary in severity [11]; additional genotype–phenotype correlation was observed for some genotypes [20].

A clinical diagnosis of SLOS generally relies on recognition of a characteristic pattern of findings. A diagnosis of SLOS is confirmed by demonstration of biallelic pathogenic *DHCR7* variants and by the detection of elevated-serum 7-DHC levels. Hypocholesterolemia is less reliable as a diagnostic parameter, as it is not present in a significant proportion of affected individuals, and because standard laboratory methods frequently determine total cholesterol together with its precursors, 7-DHC and 8-DHC [21].

We report here a pair of siblings with mild global developmental delay and infrequent epileptic seizures, and provide evidence that the homozygous *DHCR7* variant c.988G>A (p.Val330Met) underlies this phenotype. Remarkably, although 7-DHC serum levels were elevated, neither sibling displayed congenital anomalies suggestive of SLOS.

## 2. Materials and Methods

Research Human subjects: Genetic studies were approved by the Institutional Review Boards of the Medical University of Innsbruck (No. UN4501), Innsbruck, Austria. The parents provided written informed consent for their participation and that of their children in the study, with clinical data and specimen collection, genetic analysis, and publication of relevant findings including clinical images.

Serum cholesterol and 7-DHC levels were determined in the proband and affected sister as reported [22].

**Figure 1:**
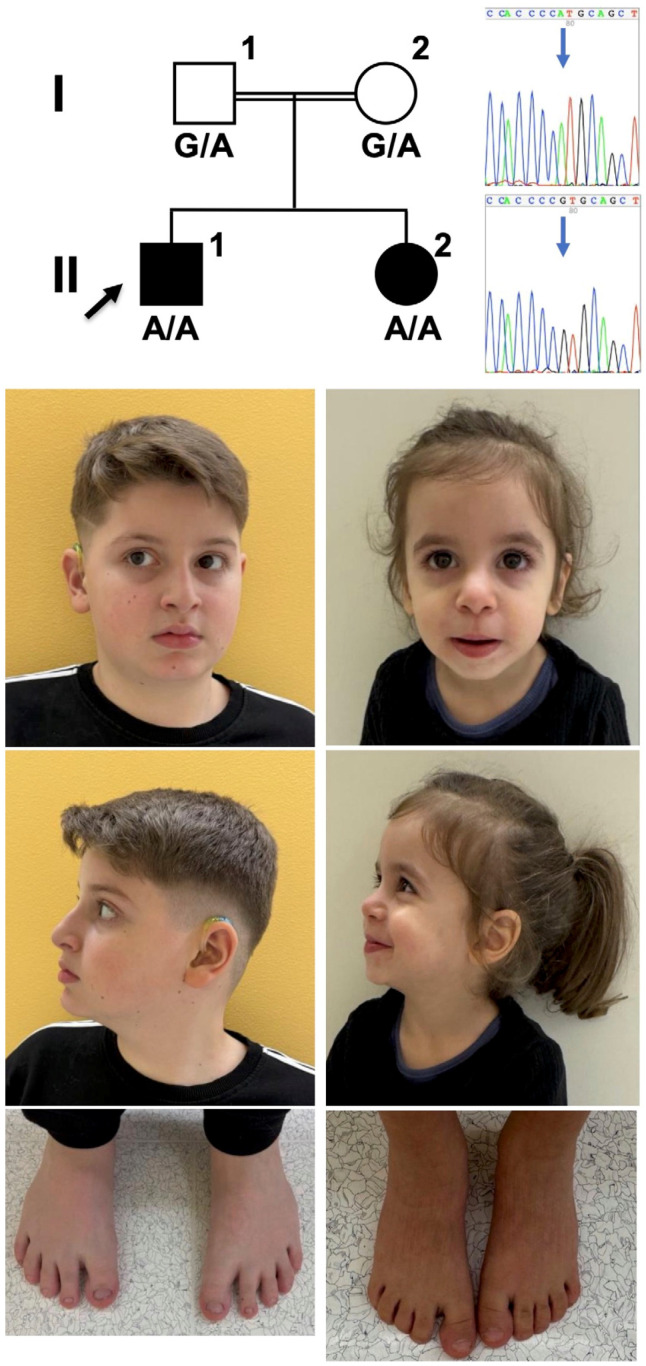
*DHCR7* variant segregation with non-syndromic ID. The patients’ parents (I-1, I-2) are cousins and heterozygotes for the *DHCR7* variant c.988G>A, their children II-1, II-2 are homozygous for this variant and display non-syndromic ID as demonstrated with photographs of frontal and lateral facial views and photographs of their feet. Some features are reminiscent of SLOS in patient II-2, i.e., a thin upper lip, a long philtrum with high insertion of the nasal columella.

Genomic DNA was extracted from peripheral blood samples using standard procedures. Exome sequencing (ES) was performed with the genomic DNA samples from the propositus, both parents, and the affected sister (Figure 1). Human protein-coding genes (36.8 Mb in total) were captured from genomic DNA with the Twist Comprehensive Exome Panel and with the Mitochondrial Panel (Twist Bioscience, San Francisco, CA, USA); reagents from the same kits were used to prepare DNA libraries, which were sequenced on a HiSeq platform (Illumina, San Diego, CA, USA) with 150 bp read length in paired-end sequencing mode. The obtained sequencing reads were aligned to the human reference genome “Genome Reference Consortium Human Build 38 Organism: Homo sapiens (GRCh38)” (University of California Santa Clara, Santa Clara, CA, USA), and single nucleotide variants and small indels were called with the Genome Analysis Toolkit (GATK) version 4.0 (https://github.com/broadinstitute/gatk, accessed on 1 February 2020). Sequencing reads were also aligned to the human reference genome “Genome Reference Consortium Human Build 37 Organism: Homo sapiens (GRCh37)” with SeqNext (Version 5.0; JSI, Kippenheim, Germany). Our GRCh37 pipeline includes single nucleotide variants and small-indel calling and the detection of single and multiple exon deletions and duplications.

Called variants were filtered for autosomal recessive mode of inheritance (including both homozygous and compound heterozygous variants) and in addition for de novo occurrence in each of the affected siblings. Variants were filtered for predicted effect on protein expression (missense, nonsense, intronic variants at exon–intron boundaries ranging from −15 to +15, in-frame indels, and frameshift), and for allele frequency of <0.01 in the gnomAD database (https://gnomad.broadinstitute.org/, accessed on 2 February 2020). Variants were evaluated in silico for pathogenicity by CADD (http://cadd.gs.washington.edu/score, accessed on 2 February 2020) [23]; missense variants were evaluated by PolyPhen-2 (http://genetics.bwh.harvard.edu/pph2, accessed on 2 February 2020) [24] and SIFT [25]; and splice site variants were evaluated using SpliceAI lookup (https://spliceailookup.broadinstitute.org/, accessed on 2 February 2020). Sanger sequencing of a genomic PCR fragment (forward primer 5′-, reverse primer 5′-) permitted *DHCR7* variant validation and segregation within the family. Variant designation is based on the National Center for Biotechnology Information reference sequence for *DHCR7* transcript NM_001360.3.

Genomic structural variants were searched in the proband by genotyping a high-resolution single nucleotide polymorphism array (HumanCytoSNP-12v2 BeadChip SNP array, Illumina), according to the manufacturer’s instructions.

## 3. Results

### 3.1. Clinical and Biochemical Findings

We report 2 sibling children with neurodevelopmental delay, born to healthy Turkish parents who are first cousins. The male proband (Figure 1, individual II-1), 10.5 years old at last examination, was born at term. Antenatal ultrasounds at 12, 20, and 35 weeks reported normal growth and development. His birth weight was 4.27 kg (>99th percentile, Z score +1.4), length was 55 cm (84th percentile, Z score +1.0), and occipitofrontal circumference was not documented. Apgar scores were 9 at 1 min and 10 at 5 min. He passed the newborn screening for inherited metabolic diseases. However, newborn screening detected bilateral sensorineural hearing loss of moderate degree, and the proband has worn hearing aids since the age of 5 months. Albeit normal hearing thresholds recorded while wearing hearing aids, he is notable for expressive language delay since age 12 months. At 2 years and 9 months, he spoke two words only. He was able to follow complex instructions when given to him repeatedly. At age 9 years, he continues to receive speech therapy for deficiencies in grammar, vocabulary, and articulation, and occupational therapy for deficits in focusing, and he attends a school for children with particular needs, with first-year schooling material. Formal developmental testing at age 9 years using the Wechsler Intelligence Scale for Children (WISC-V), the Development Test of Visual-Motor Integration (VMI) including a subtest for visual perception, and questionnaire CBCL 6–18 revealed an average intelligence quotient (IQ) score of 79 points (range 75–85 points; reference 85–114 points). Since early childhood he is irritable and displays aggressive and auto-aggressive behavior with difficulties in social interactions and a reduced attention span for his age, as well as bruxism. The patient experienced focal epileptic seizures, first noted at age 7 years, which responded to mono-therapy with lamotrigine for 2 years and did not recur within the last 18 months. Electroencephalogram demonstrated a Rolando focus. He takes melatonin per os since age 5 years for markedly reduced sleep duration, fragmented sleep, and difficulty falling asleep. He thrives at the 99th percentile (Z score +2.42), with type 1 obesity (BMI 26.8, >99th percentile, Z score +2.38), and proportionate head circumference of 56 cm (94th percentile, Z score +1.59). Clinical examination was unremarkable, and typical facial features of SLOS such as down-slanting palpebral fissures, broad nasal bridge, low-set and prominent ears, bilateral single transverse palmar creases, pectus excavatum, and bilateral 2–3 toe syndactyly were all absent (Figure 1). The patient underwent the calcaneo-stop procedure for flexible flatfoot. Phenotypic severity, based on the degree and number of physical malformations, was determined to be 5 (reference: mild < 20, moderate 20–50, severe > 50) using the SLOS physical severity score [10]. The SLOS severity score does not consider the severity of ID, behavioral disturbances, or feeding difficulties. A cranial MRI at age 9.5 years was normal.

Biochemical testing for SLOS was performed at 10 years of age following the identification of a rare homozygous *DHCR7* variant through exome sequencing (ES). Levels of 7-DHC (3.9 µmol/L, ref. 0.4–1.5 µmol/L) and 8-DHC (5.1 µmol/L, ref. 0.1–1.2 µmol/L) were elevated, and cholesterol was within normal range (3.8 mmol/L, ref. 2–5 mmol/L). A chromosomal array was normal. Thyroid function, growth hormone, cortisol, and adrenocorticotropic hormone were all reported as within normal range. The patient displays mildly decreased serum plasminogen activity (49%, ref. 75–150%) without apparent clinical signs, recognized during family-based, unrelated targeted testing for plasminogen deficiency.

The proband’s younger sister (II-2), now 3 years old, came to medical attention first at 3 months of age with protracted diarrhea and failure to thrive, which led to a diagnosis of very-early-onset inflammatory bowel disease (IBD) following gastroduodenoscopy with demonstration of inflammation in her intestinal biopsies. At age 4 months she presented with ligneous conjunctivitis as a result of plasminogen deficiency. The girl was born at term after uneventful pregnancy weighing 3230 g (43rd percentile, Z −0.17), length 52 cm (65th percentile, Z +0.39), and head circumference of 35 cm (78th percentile, Z +0.76). The IBD responded to immunosuppression with prednisolone, azathioprine, and adalimumab, and the patient thrives at the 25th percentile, with a head circumference of 48 cm (16th percentile). The patient displays mild global developmental delay, learned to walk at 21 months, and currently features a vocabulary of fewer than 20 words. She displays an obtrusive behavior and non-compliance to requests, leading to a diagnosis of mild ID. Seizures occurred twice. Clinical examination was otherwise unremarkable, and typical facial features of SLOS, 2–3 toe syndactyly, and malformations were all absent (SLOS score of 5 (reference: mild < 20, moderate 20–50, severe > 50). Levels of 7-DHC (4.4 µmol/L, ref. 0.4–1.5 µmol/L), 8-DHC (4.8 µmol/L, ref. 0.1–1.2 µmol/L) and cholesterol were mildly elevated (5.4 mmol/L, ref. 2–5 mmol/L). The patient displays varying levels of decreased serum plasminogen activity (30–53%, ref. 75–150%) with symptoms of plasminogen deficiency.

### 3.2. Molecular Findings

Chromosomal microarray analysis in the proband was normal. ES in the propositus revealed homozygosity for *DHCR7* variant c.988G>A (p.Val330Met), residing within 32.8 Mbps of homozygosity. This variant (rs139724817) is listed in the gnomAD population database v4.1.0 with an overall allele frequency of 659 in 1,603,764 (0.04%), and an allele frequency of 0.25% in Middle Eastern individuals, in heterozygous state only. The variant affects a highly conserved amino acid, and is predicted to affect protein function in silico by CADD, PolyPhen-2, and SIFT algorithms. Homozygosity for this variant, localized within 26.5 Mbps of homozygous sequence, was also found in the sister, and both parents are heterozygotes; Sanger sequencing showed variant segregation with the phenotype of mild ID, infrequent seizures, elevated serum 7-DHC, and absence of characteristic SLOS dysmorphism and malformations in the extended family (Figure 1).

Maternal lipoprotein *APOE* [26] and *ABCA1* [27] variants known to increase the clinical severity of SLOS were not present in ES data of the patients’ mother.

## 4. Discussion

We report a pair of siblings with mild, non-syndromic ID and with infrequent seizures in whom ES identified the homozygous *DHCR7* variant c.988G>A. This variant was localized within large regions of homozygosity in both siblings, consistent with inheritance identical-by-descent, and segregated with disease in the family. Of note, ES did not identify other pathogenic or likely pathogenic variants that explained the ID in each sibling. Elevated serum 7-DHC levels in these patients indicated that the c.988G>A variant affected 7-DHC reductase function and was therefore classified as pathogenic. This variant had been considered as pathogenic once before in compound-heterozygous state with a p.Arg363Cys variant in an 8-year-old female patient with a diagnosis of SLOS (severity score of 26, moderate SLOS) and normal serum 7-DHC levels [28]. The novel finding in our report is the absence of any dysmorphic features suggestive of SLOS in our patients with mild ID; moreover, neither of the siblings we report here had short stature or microcephaly; the proband’s length and BMI were above the 97th percentile, which is a very atypical finding in SLOS [29]. On the other hand, the behavioral abnormalities, in particular, autistic features [17], the seizures, and the otherwise unexplained sensorineural hearing loss in the proband fit well into the SLOS spectrum [8,9].

Our observation leads us to conclude that *DHCR7* variant c.988G>A, and potentially other *DHCR7* variants, might be considered as underlying cases of non-syndromic ID. Homozygosity for the c.988G>A variant might lead to mild SLOS with typical facial features or congenital malformations as well, given the inter- and intrafamilial variability observed with other *DHCR7* genotypes. To consider *DHCR7* variants to underlie non-specific ID was previously suggested. One study recognized mild SLOS in 5 patients with mild ID, albeit all 5 patients also displayed the typical 2–3 toe syndactyly, displayed microcephaly, and had facial features suggestive of SLOS and feeding problems in childhood [12]. ID with minimal physical signs of SLOS was further reported in three related patients, in whom the diagnosis of SLOS was delayed for years [30]. Mild phenotypes of SLOS with respect to cognitive function were elsewhere reported but generally featured 2–3 toe syndactyly, feeding difficulties, failure to thrive, and elevated plasma 7-DHC [14,31,32,33]. One study reported normal or low-normal IQ (children with at least one IQ composite score above 80) with elevated serum 7-DHC levels in six girls and three boys from a cohort of 145 children with SLOS. No correlation with IQ and genotype was evident, and no specific developmental profile were observed. Major and multiple organ anomalies were uncommon, but minor anomalies were suggestive of SLOS [13].

Because of their poor expressive language and hyperactivity, developmental testing might underestimate the cognitive abilities in such patients. Gross motor development is typically more severely delayed than fine motor development, but most children with classical SLOS learn to walk between 2 and 4 years [8,17].

## 5. Conclusions

Our study indicates that decreased 7-DHC reductase activity might underlie low-normal IQ and mild ID presenting as apparently non-syndromic global developmental delay.

## Data Availability

All data relating to this study are contained in this report. The exome sequencing dataset of the index patient is unavailable due to privacy restrictions.
